# Supplementary material for: Heat adaptation of phage T7 under an extended genetic code
Source: Virus Evol. 2021 Dec 1;7(2):veab100. doi: 10.1093/ve/veab100 (PMC8923235; doi:10.1093/ve/veab100)
Supplement: veab100_Supp [file veab100_supp.zip › Sequencing_Analysis.zip!/Sequencing_Analysis/S3/evidence/SNP_14.html]

BRESEQ :: Evidence


| Predicted mutation | | | | | | |
| --- | --- | --- | --- | --- | --- | --- |
| evidence | seq id | position | mutation | annotation | gene | description |
| RA | NC\_001604.1 | 32,079 | A→C | intergenic (–/–) | – / – | –/– |

| Read alignment evidence... | | | | | | | | | | | |
| --- | --- | --- | --- | --- | --- | --- | --- | --- | --- | --- | --- |
|  | seq id | position | | ref | new | freq | score (cons/poly) | reads | annotation | genes | product |
| \* | NC\_001604.1 | 32,079 | 0 | A | C | 82.9% | 3918.1 / inf | 1708 | intergenic (–/–) | *–/–* | –/– |
| Reads supporting (aligned to +/- strand):  **ref** base A (159/133);  **new** base C (715/698);  **total** (875/833) | | | | | | | | | | | |
| Fisher's exact test for biased strand distribution *p*-value = 2.47e-01 | | | | | | | | | | | |
| Kolmogorov-Smirnov test that lower quality scores support variant *p*-value = 9.90e-01 | | | | | | | | | | | |

Only 100 of 1742 total aligned reads displayed.  

`GTGTTTGGCGCAGGCATGTCTGCAATCAGTGACGCTGTAGCTGCTGGACTGAAACGCAGTAAACCAGAAGCTGAGTTCGACAATGAGTTCATCGGTCCTATGATGCGATTGGAAGCCCGTGAGACAGCACGAAACGCCAACTCTGCGGACCTCTCTCGGATGAACACTGAGAACATGAAGTTTGAAGGTGAACATAATGGTGTCCCTTATGAGGACTTACCAACAGAGAGAGGTGCCGTGGTGTTACATGATGGCTCCGTTCTAAGTGCAAGCAACCCAATCAACCCTAAGACTCTAAAAGAGTTCTCCGAGGTTGACCCTGAGAAGGCTGCGCGAGGAATCAAACTGGCT‑GGGTTCACCGAGATTGGCTTGAAGACCTTGGGGTCTGACGATGCTGACATCCGTAGAGTGGCTATCGACCTCGTTCGCTCTCCTACTGGTATGCAGTCTGGTGCCTCAGGTAAGTTCGGTGCAACAGCTTCTGACATCCATGAGAGACTTCATGGTACTGACCAGCGTACTTATAATGACTTGTACAAAGCAATGTCTGACGCTATGAAAGACCCTGAGTTCTCTACTGGCGGCGCTAAGATGTCC  >  NC_001604.1/31780‑32376`  

                                                                                                                                                                                                                                                                                                           `|`                                                                                                                                                                                                                                                                                                            
`gtgtTTGGCGCAGGCATGTCTGCAATCAGTGACGCTGTAGCTGCTGGACTGAAACGCAGTAAACCAGAAGCTGAGTTCGACAATGAGTTCATCGGTCCTATGATGCGATTGGAAGCCCGTGAGACAGCACGAAACGCCAACTCTGCGGACCTCTCTCGGATGAACACTGATAACATGAAGTTTGAAGGTGAACATAATGGTGTCCCTTATGAGGACTTACCAACAGAGAGAGGTGCCGTGGTGTTACATGATGGCTCCGTTCTAAGTGCAAGCAACCCAATCAACCCTAAGACTCTAAAag                                                                                                                                                                                                                                                                                                           >  1:174170/1‑301 (MQ=255)`  
       `gcgcAGGCATGTCTGCAATCAGTGACGCTGTAGCTGCTGGACTGAAACGCAGTAAACCAGAAGCTGAGTTCGACAATGAGTTCATCGGTCCTATGATGCGATTGGAAGCCCGTGAGACAGCACGAAACGCCAACTCTGCGGACATCTCTCGGATGAACACTGAGAACATGAAGTTTGAAGGTGAACATAATGGTGTCCCTTATGAGGACTTACCAACAGAGAGAGGTGCCGTGGTGTTACATGATGGCTCCGTTCTAAGTGCAAGCAACCCAATCAACCCTAAGACTCTAAACGAGTtctc                                                                                                                                                                                                                                                                                                    <  1:295985/301‑1 (MQ=255)`  
             `gCATGTCTGCAATCAGTGACGCTGTAGCTGCTGGACTGAAACGCAGTAAACCAGAAGCTGAGTTCGACAATGAGTTCATCGGTCCTATGATGCGATTGGAAGCCCGTGAGACAGCACGAAACGCCAACTCTGCGGACCTCTCTCGGATGAACACTGAGAACATGAAGTTTGAAGGTGAACATAATGGTGTCCCTTATGAGGACTTACCAACAGAGAGAGGTGCCGTGGTGTTACATGATGGCTCCGTTCTGAGTGCAAGCAACCCAATCAACCCTAAGACTCTAAACGAGTTCTCCGAGGt                                                                                                                                                                                                                                                                                              <  1:302128/301‑1 (MQ=255)`  
                 `ttCTGCAATCAGTGACGCTGTAGCTGCTGGACTGAAACGCAGTAAACCAGAAGCTGAGTTCGACAATGAGTTCATCGGTCCTATGATGCGATTGGAAGCCCGTGAGACAGCACGAAACGCCAACTCTGCGGACCTCTCTCGGATGAACACTGAGAACATGAAGTTTGAAGGTGAACATAATGGTGTCCCTTATGAGGACTTACCAACAGAGAGAGGTGCCGTGGTGTTACATGATGGCTCCGTTCTAAGTGCAAGCAACCCAATCAACCCTAAGACTCTAAACGAGTTCTCCGAGGTTGAc                                                                                                                                                                                                                                                                                          <  1:236466/300‑1 (MQ=255)`  
                 `gTCTGCAATCAGTGACGCTGTAGCTGCTGGACTGAAACGCAGTAAACCAGAAGCTGAGTTCGACAATGAGTTCATCGGTCCTATGATGCGATTGGAAGCCCGTGAGACAGCACGAAACGCCAACTCTGCGGACCTCTCTCGGATGAACACTGAGAACATGAAGTTTGAAGGTGAACATAATGGTGTCCCTTATGAGGACTTACCAACAGAGAGAGGTGCCGTGGTGTTACATGATGGCTCCGTTCTAAGTGCAAGCAACCCAATCAACCCTAAGACTCTAAACGAGTTCTCCGAGGTTGAc                                                                                                                                                                                                                                                                                          >  1:241508/1‑301 (MQ=255)`  
                        `aTCAGTGACGCTGTAGCTGCTGGACTGAAACGCAGTAAACCAGAAGCTGAGTTCGACAATGAGTTCATCGGTCCTATGATGCGATTGGAAGCCCGTGAGACAGCACGAAACGCCAACTCTGCGGACCTCTCTCGGATAAACACTGAGAACATGAAGTTTGAAGGTGAACATAATGGTGTCCCTTATGAGGACTTACCAACAGAGAGAGGTGCCGTGGTGTTACATGATGGCTCCGTTCTAAGTGCAAGCAACCCAATCAACCCTAAGACTCTAAAAGAGTTCTCCGAGGTTGACCCTgaga                                                                                                                                                                                                                                                                                   >  1:140413/1‑301 (MQ=255)`  
                          `cgtgggctcggagatgtgtataagagacagCAGTAAACCAGAAGCTGAGTTCGACAATGAGTTCATCGGTCCTATGATGCGATTGGAAGCCCGTGAGACAGCACGAAACGCCAACTCTGCGGACCTCTCTCGGATGAACACTGAGAACATGAAGTTTGAAGGTGAACATAATGGTGTCCCTTATGAGGACTTACAAACAGAGAGAGGTGCCGTGGTGTTACATGATGGCTCCGTTCTAAGTGCAAGAAACCCAATCAACCCTAAGACTCTAAACGAGTTCTCCGAGGTTGACCCTGAGAAg                                                                                                                                                                                                                                                                                 <  1:264911/272‑1 (MQ=255)`  
                          `cAGTGACGCCGTAGCTGCTGGACTGAAACGCAGTAAACCAGAAGCTGAGTTCGACAATGAGTTCATCGGTCCTATGATGCGATTGGAAGCCCGTGAGACAGCACGAAACGCCAACTCTGCGGACCTCTCTCGGATGAACACTGAGAACATGAAGTTTGAAGGTGAACATAATGGTGTCCCTTATGAGGACTTACCAACAGAGAGAGGTGCCGTGGTGTTACATGATGGCTCCGTTCTAAGTGCAAGCAACCCAATCAACCCTAAGACTCTAAAcgagctgtctcttatacacatctccgag                                                                                                                                                                                                                                                                                 >  1:32138/1‑277 (MQ=255)`  
                                  `cTGTAGCTGCTGGACTGAAACGCAGTAAACCAGAAGCTGAGTTCGACAATGAGTTCATCGGTCCTATGATGCGATTGGAAGCCCGTGAGACAGCACGAAACGCCAACTCTGCGGACCTCTCTCGGATGAACACTGAGAACATGAAGTTTGAAGGTGAACATAATGGTGTCCCTTATGAGGACTTACCAACAGAGAGAGGTGCCGTGGTGTTACATGATGGCTCCGTTCTAAGTGCAAGCAACCCAATCAACCCTAAGACTCTAAACGAGTTCTCCGAGGTTGACCCTGAGAAGGCTGcgcg                                                                                                                                                                                                                                                                         >  1:115891/1‑301 (MQ=255)`  
                                  `cTGTAGCTGCTGGACTGAAACGAAGTAAACCAGAAGCTGAGTTCGACAATGAGTTCATCGGTCCTATGATGCGATTGGAAGCCCGTGAGACAGCACGAAACGCCAACTCTGCGGACCTCTCTCGGATGAACACTGAGAACATGAAGTTTGAAGGTGAACATAATGGTGTCCCTTATGAGGACTTACCAACAGAGAGAGGTGCCGTGGTGTTACATGATGGCTCCGTTCTAAGTGCAAGCAACCCAATCAACCCTAAGACTCTAAAAGAGTTCTCCGAGGTTGACCCTGAGAAGGCTGcgcg                                                                                                                                                                                                                                                                         <  1:234085/301‑1 (MQ=255)`  
                                                      `cgaaGTAACCCAGAAGCTGAGTTCGACAATGAGTTCATCGGTCCTATGATGCGATTGGAAGCCCGTGAGACAGCACGAAACGCCAACTCTGCGGACCTCTCTCGGATGAACACTGAGAACATGAAGTTTGAAGGTGAACATAATGGTGTCCCTTATGAGGACTTACCAACAGAGAGAGGTGCCGTGGTGTTACATGATGGCTCCGTTCTAAGTGCAAGCAACCCAATCAACCCTAAGACTCTAAACGAGTTCTCCGAGGTTGACCCTGAGAAGGCTGCGCGAGGAATCAAACTGGCT‑GGGt                                                                                                                                                                                                                                                    <  1:88916/298‑1 (MQ=255)`  
                                                                        `gAGTTCGACAATGAGTTCATCGGTCCTATGATGCGATTGGAAGCCCGTGAGACAGCACGAAACGCCAACTCTGCGGACCTCTCTCGGATGAACACTGAGAACATGAAGTTTGAAGGTGAACATAATGGTGTCCCTTATGAGGACTTACCAACAGAGAGAGGTGCCGTGGTGTTACATGATGGCTCCGTTCTAAGTGCAAGCAACCCAATCAACCCTAAGACTCTAAACGAGTTCTCCGAGGTTGACCCTGAGAAGGCTGCGCGAGGCATCAAACTGGCT‑GGGTTCACCGAGATTGTCTTGa                                                                                                                                                                                                                                  >  1:64203/1‑301 (MQ=255)`  
                                                                                   `tGAGTTCATCGGTCCTATGATGCGATTGGAAGCCCGTGAGACAGCACGAAACGCCAACTCTGCGGACCTCTCTCGGATGAACACTGAGAACATGAAGTTTGAAGGTGAACATAATGGTGTCCCTTATGAGGACTTACCAACAGAGAGAGGTGCCGTGGTGTTACATGATGGCTCCGTTCTAAGTGCAAGCAACCCAATCAACCCTAAGACTCTAAACGAGTTCTCCGAGGTTGACCCTGAGAAGGCTGCGCGAGGAATCAAACTGGCT‑GGGTTCACCGAGATTGGCTTGAAGACCTTgggg                                                                                                                                                                                                                       <  1:48265/301‑1 (MQ=255)`  
                                                                                                `ccTATGATGCGATTGGAAGCCCGTGAGACAGCACGAAACGCCAACTCTGCGGACCTCTCTCGGATGAACACTGAGAACATGAAGTTTGAAGGTGAACATAATGGTGTCCCTTATGAGGACTTACCAACAGAGAGAGGTGCCGTGGTGTTACATGATGGCTCCGTTCTAAGTGCAAGCAACCCAATCAACCCTAAGACTCTAAACGAGTTCTCCGAGGTTGACCCTGAGAAGGCTGCGCGAGGAATCAAACTGGCT‑GGGTTCACCGAGATTGGCTTGAAGACCTTGGGGTCTGACGATGCTg                                                                                                                                                                                                          >  1:11274/1‑301 (MQ=255)`  
                                                                                                          `gATTGGAAGCCCGTGAGACAGCACGAAACGCCAACTCTGCGGACCTCTCTCGGATGAACACTGAGAACATGAAGTTTGAAGGTGAACATAATGGTGTCCCTTATGAGGACTTACCAACAGAGAGAGGTGCCGTGGTGTTACATGATGGCTCCGTTCTAAGTGCAAGCAACCCAATCAACCCTAAGACTCTAAACGAGTTCTCCGAGGTTGACCCTGAGAAGGCTGCGCGAGGAATCAAACTGGCT‑GGGTTCACCGAGATTGGCTTGAAGACCTTGGGGTCTGACGATGCTGACATCCGTag                                                                                                                                                                                                <  1:145974/301‑1 (MQ=255)`  
                                                                                                            `ttGGAAGCCCGTGAGACAGCACGAAACGCCAACTCTGCGGACCTCTCTCGGATGAACACTGAGGACATGAAGTTTGAAGGTGAACATAATGGTGTCCCTTATGAGGACTTACCAACAGAGAGAGGTGCCGTGGTGTTACATGATGGCTCCGTTCTAAGTGCAAGCAACCCAATCAACCCTAAGACTCTAAACGAGTTCTCCGAGGTTGACCCTGAGAAGGCTGCGCGAGGAATCAAACTGGCT‑GGGTTCACCGAGATTGGCTTGAAGACCTTGGGGTCTGACGATGCTGACATCCGTagag                                                                                                                                                                                              <  1:97949/301‑1 (MQ=255)`  
                                                                                                             `tGGAAGCCCGTGAGACAGCAAGAAACGCCAACTCTGCGGACCTCTCTCGGATGAACACTGAGAACATGAAGTTTGAAGGTGAACATAATGGTGTCCCTTATGAGGACTTACGAACAGAGAGAGGTGCCGTGGTGTTACAGGATGGATCCGTTCTAAGTGCAAGCAACCCAAGCAACCCTAAGACTCTAAACGAGTTCTCCGAGGTTGACCCTGAGAAGGCTGCGCGAGGAATCAAACTGGCT‑GGGTTCACCGAGATTGGCTTGAAGACCTTGGGGTCTGACGATGCTGACATCCGTAGAGt                                                                                                                                                                                             <  1:346374/301‑1 (MQ=255)`  
                                                                                                                       `gagatgtgtataagagacagaCTCTGCGGACCTCTCTCGGATGAACACTGAGAACATGAAGTTTGAAGGTGAACATAATGGTGTCCCTTATGAGGACTTACCAACAGAGAGAGGTGCCGTGGTGTTACATGATGGCTCCGTTCTAAGTGCAAGCAACCCAATCAACCCTAAGACTCTAAACGAGTTCTCCGAGGTTGACCCTGAGAAGGCTGCGCGAGGAATCAAACTGGCT‑GGGTTCACCGAGATTGGCTTGAAGACCTTGGGGTCTGACGATGCTGACATCCGTAGAGTGGCTATCGAc                                                                                                                                                                                   <  1:238666/281‑1 (MQ=255)`  
                                                                                                                        `aagatgggtataagagacagctctGCGGACCTCTCTCGGATGAACACTGAGAACATGAAGTTTGAAGTTGAACATAATGGTGTCCCTTATGAGGACTTACCAACAGAGAGAGGTGCCGTGGTGTTACATGATGGCTCCGTTCTAAGTGCAAGCAACCCAATCAACCCTAAGACTCTAAACGAGATCTCCGAGGTTGACCCTGAGAAGGCTGCGCGAGGAATCAAACTGGCT‑GGGTTCACCGAGATTGGCTTGAAGACCTTGGGGTCTGACGATGCTGACATCCGTAGAGTGGCTATCGAcc                                                                                                                                                                                  <  1:5556/281‑1 (MQ=255)`  
                                                                                                                        `aagaCAGCACGAAACGCCAACTCTGCGGACCTCTCTCGGATGAACACTGAGAACATGAAGTTTGAAGGTGAACATAATGGTGTCCCTTATGAGGACTTACCAACAGAGAGAGGTGCCGTGGTGTTACATGATGGCTCCGTTCTAAGTGCAAGCAACCCAATCAACCCTAAGACTCTAAACGAGTTCTCCGAGGTTGACCCTGAGAAGGCTGCGCGAGGAATCAAACTGGCT‑GGGTTCACCGAGATTGGCTTGAAGACCTTGGGGTCTGACGATGCTGACATCCGTAGAGTGGCTATCGAcc                                                                                                                                                                                  <  1:244640/300‑1 (MQ=255)`  
                                                                                                                         `agaCAGCACGAAACGCCAACTCTGCGGACCTCTCTCGGATGAACACTGAGAACATGAAGTTTGAAGGTGAACATAATGGTGTCCCTTATGAGGACTTACCAACAGAGAGAGGTGCCGTGGTGTTACATGATGGCTCCGTTCTAAGTGCAAGCAACCCAATCAACCCTAAGACTCTAAACGAGTTCTCCGAGGTTGACCCTGAGAAGGCTGCGCGAGGAATCAAACTGGCT‑GGGTTCACCGAGATTGGCTTGAAGACCTTGGGGTCTGACGATGCTGACATCCGTagagctgtctcttatac                                                                                                                                                                                 >  1:259900/1‑288 (MQ=255)`  
                                                                                                                                          `aaCTCTGCGGACCTCTCTCGGATGAACACTGAGAACATGAAGTTTGAAGGTGAACATAATGGTGTCCCTTATGAGGACTTACCAACAGAGAGAGGTGCCGTGGTGTTACATGATGGCTCCGTTCTAAGTGCAAGCAACCCAATCAACCCTAAGACTCTAAACGAGTTCTCCGAGGTTGACCCTGAGAAGGCTGCGCGAGGAATCAAACTGGCT‑GGGTTCACCGAGATTGGCTTGAAGACCTTGGGGTCTGACGATGCTGACATCCGTAGAGTGGCTATCGACCTCGTTCGCTCTCCTACTg                                                                                                                                                                >  1:105577/1‑301 (MQ=255)`  
                                                                                                                                           `aCTCTGCGGACCTCTCTCGGATGAACACTGAGAACATGAAGTTTGAAGGTGAACATAATGGTGTCCCGTATGAGGACTTACCAACAGAGAGAGGTGCCGTGGTGTTACATGATGGCTCCGTTCTAAGTGCAAGCAACCCAATCAACCCTAAGACTCTAAACGAGTTCTCCGAGGTTGACCCTGAGAAGGCTGCGCGAGGAATCAAACTGGCT‑GGGTTCACCGAGATTGGCTTGAAGACCTTGGGGTCTGACGATGCTGACATCCGTAGAGTGGCTATCGACCTCATTCGCTCTCCTACTgg                                                                                                                                                               <  1:161468/301‑1 (MQ=255)`  
                                                                                                                                            `ctctGCGGACCTCTCTCGGATGAACACTGAGAACATGAAGTTTGAAGGTGAACATAATGGTGTCCCTTATGAGGACTTACCAACAGAGAGAGGTGCCGTGGTGTTACATGATGGCTCCGGTCTAAGTGCAAGCAACCCAATCAACACTAAGACTCTAAAAGAGTTCTCCGAGGTTGACCCTGAGAAGGCTGCGCGAGGAATCAAACTGGCT‑GGGTTCACCGAGCTTGGCTTGAAGACCTTGGGGTCTGACGCTGCTAACCTCCGTAGAGTGGCTATCGACCTCGTTCGCTCTCCTACTGGt                                                                                                                                                              >  1:92167/1‑301 (MQ=255)`  
                                                                                                                                              `ctGCGGACCTCTCTCGGATGAACACTGAGAACATGAAGTTTGAAGGTGAACATAATTGTGTCCCTTATGAGGACTTACCAACAGAGAGAGGTGCCGTGGTGTTACATGATGGCTCCGTTCTAAGTGCAAGCAACCCAATCAACCCTAAGACTCTAAAAGAGTTCTCCGAGGTTGACCCTGAGAAGGCTGCGCGAGGAATCAAACTGGCT‑GGGTTCACCGAGATTGGCTTGAAGACCTTGTGGTCTGACGATGCTGACATCCGTAGAGTGGCTATCGACCTCCtttctcttatacacatctc                                                                                                                                                            >  1:132196/1‑284 (MQ=255)`  
                                                                                                                                                     `ccTCTCTCGGATGAACACTGAGAACATGAAGTTTGAAGGTGAACATAATGCTGTCCCTTATGAGGACTTACCAACAGAGAGATGTGCCGTGGTGTTACATGATGGCTCCGTTCTAAGTGCAAGCAACCCAATCAACCCTAAGACTCTAAACGAGTTCTCCGAGGTTGACCCTGAGAAGGCTGCGCGCGGAATCAAACTGGCT‑GGGTTCACCGAGATTGGCTTGAAGACCTTGGGGTCTGACGATGCTGCCATCCGTAGAGTGGCTATCGACCTCGTTCGCTCTCCTACTGGTATGCAGTCt                                                                                                                                                     >  1:38684/1‑301 (MQ=255)`  
                                                                                                                                                              `gATGAACACTGAGAACATGAAGTTTGAAGGTGAACATAATGGTGTCCCTTATGAGGACTTACCAACAGAGAGAGGTGCCGTGGTGTTACATGATGGCTCCGTTCTAAGTGCAAGCAACCCAATCAACCCTAAGACTCTAAACGAGTTCTCCGAGGTTGACCCTGAGAAGGCTGCGCGAGGAATCAAACTGGCT‑GGGTTCACCGAGATTGGCTTGAAGACCTTGGGGTCTGACGATGCTGACATCCGTAGAGTGGCTATCGACCTCGTTCGCTCTCCTACTGGTATGCAGTCTGGTGCCTCa                                                                                                                                            >  1:138523/1‑301 (MQ=255)`  
                                                                                                                                                                  `aaCACTGAGAACATGAAGTTTGAAGGTGAACATAATGGTGTCCCTTATGAGGACTTACCAACAGAGAGAGGTGCCGTGGTGTTACATGATGGCTACGTTCTAAGTGCAAGCAACCCAATCAACCCTAAGACTCTAAACGAGTTCTCCGAGGTTGACCCTGAGAAGGCTGCGCGAGGAATCAAACTGGCT‑GGGTTCACCGAGATTGGCTTGAAGACCTTGGGGTCTGACGATGCTGACATCCGTAGAGTGGCTATCGACCTCGTTCGCTCTCCTACTGGTATGCAGTCTGGTGCCTCAGGTa                                                                                                                                        <  1:25720/301‑1 (MQ=255)`  
                                                                                                                                                                      `cTGAGAACATGAAGTTTGAAGGTGAACATAATGGTGTCCCTTATGAGGACTTACCAACAGAGAGAGGTGCCGTGGTGTTACATGATGGCTCCGTTCTAAGTGCAAGCAACCCAATCAACCCTAAGACTCTAAACGAGTTCTCCGAGGTTGACCCTGAGAAGGCTGCGCGAGGAATCAAACTGGCT‑GGGTTCACCGAGATTGGCTTGAAGACCTTGGGGTCTGACGATGCTGACATCCGTAGAGTGGCTATCGACCTCGTTCGCTCTCCTACTGGTATGCAGTCTGGTGCCTCAGGTAAGtt                                                                                                                                    <  1:250809/301‑1 (MQ=255)`  
                                                                                                                                                                       `ctcggagatgtgtataagagacagaCATAATGGTGTCCCTTATGAGGACTTACCAACAGAGAGAGGTGCCGTGGTGTTACATGATGGCTCCGTTCTAAGTGCAAGCAACCCAATCAACCCTAAGACTCTAAACGAGTTCTCCGAGGTTGACCCTGAGAAGGCTGCGCGAGGAATCAAACTGGCT‑GGGTTCACCGAGATTGGCTTGAAGACCTTGGGGTCTGACGATGCTGACATCCGTAGAGTGGCTATCGACCTCGTTCGCTCTCCTACTGGTATGCAGTCTGGTGCCTCAGGTAAGTTc                                                                                                                                   <  1:34026/277‑1 (MQ=255)`  
                                                                                                                                                                          `gaACATGAAGTTTGAAGGTGAACATAATGGTGTCCCTTATGAAGACTTACAAACAGAGAGAGGTGCCGTGGTGTTACATGATGGCTCCGTTCTAAGTGCAAGCAACCCAATCAACCCTAAGACTCTAAACGAGTTCTCCGAGGTTGACCCTGAGAAGGCTGCGCGAGGAATCAAACTGGCT‑GGGTTCACCGAGATTGGCTTGAAGACCTTGGGGTCTGACGATGCTGACATCCGTAGAGTGGCTATCGACCTCGGTCGCTCTCCTACTGGTATGCAGTCTGGTGCCTCAGGTAAGTTCGGt                                                                                                                                >  1:197831/1‑301 (MQ=255)`  
                                                                                                                                                                           `aaCATGAAGTTTGAAGGTGAACATAATGGTGTCCCTTATGAGGACTTACCAACAGAGAGAGGTGCCGTGGTGTTACATGATGGCTCCGTTCTAAGTGCAAGCAACCCAATCAACCCTAAGACTCTAAACGAGTTCTCCGAGGTTGACCCTGAGAAGGCTGCGCGAGGAATCAAACTGGCT‑GGGTTCACCGAGATTGGCTTGAAGACCTTGGGGTCTGACGATGCTGACATCCGTAGAGTGGCTATCGACCTCGTTCGCTCTCCTACTGGTATGCAGTCTGGTGCCTCAGGTAAGTTCGGTg                                                                                                                               >  1:327470/1‑301 (MQ=255)`  
                                                                                                                                                                                   `gTTTGAAGGTGAACATAATGGTGTCCCTTATGAGGACTTACCAACAGAGAGAGGTGCCGTGGTGTTACATGATGGCTCCGTTCTAAGTGCAAGCAACCCAATCAACCCTAAGACTCTAAACGAGTTCTCCGAGGTTGACCCTGAGCAGGCTGCGCGAGGAATCAAACTGGCT‑GGGTTCACCGAGATTGGCTTGAAGACCTTGGGGTCTGACGTTGCTGACATCCGTAGAGTGGCTATCGACCTCGTTCGCTCTCCTACTGGTATGCAGTCTGGTGCCTCAGGTAAGTTCGGTGCAACAGCt                                                                                                                       >  1:177275/1‑301 (MQ=255)`  
                                                                                                                                                                                   `gTTTGAAGGTGAACATAATGGTGTCCCTTATGAGGACTTACCAACAGAGAGAGGTGCCGTGGTGTTACATGATGGCTCCGTTCTAAGTGCAAGCAACCCAATCAACCCTAAGACTCTAAACGAGTTCTCCGAGGTTGACCCTGAGAAGGCTGCGCGAGGAATCAAACTGGCT‑GGGTTCACCGAGATTGGCTTGAAGACCTTGGGGTCTGACGATGCTGACATCCGTAGAGTGGCTATCGACCTCGTTCGCTCTCCTACTGGTATGCAGTCTggcggtatcttatacacatgtccgagcccc                                                                                                                       >  1:248709/1‑273 (MQ=255)`  
                                                                                                                                                                                     `gacaaGGTGAACAGAATGGTGTCCCTTATGAGGACTTAACAACAGAGGGAGGTGCCGTGGTGTTACATGAAGGCTCCGGTCTAAGGTCAAGCAACCCAATCAACCCTAAGACTCTAAACGAGTTCTCCGAGGTTGACCCTGAGAAGGCTGCGAGAGGAATCAAACTGGCTGGGGATCACCGAGATTGGCTTGAAGAACTTGGGGTCTGACGATGCTGACAGCCGTAGAGTGGCTATCGACCTCGTTCGCTCTCCTACTGGTATGCAGTCTGGTGCCTCAGGTAAGTTCGGTGCAACAGCtt                                                                                                                      <  1:183592/298‑1 (MQ=255)`  
                                                                                                                                                                                         `aagagacagtAAGGGTGTCCCTTATGAGGACTTACCAACAGAGAGAGGTGCCGTGGTGTTACATGATGGCTCCGTTCTAAGTGCAAGCAACCCAATCAACCCTAAGACTCTAAACGAGTTCTCCGAGGTTGACCCTGAGAAGGCTGCGCGAGGAATCAAACTGGCT‑GGGTTCACCGAGATTGGCTTGAAGACCTTGGGGTCTGACGATGCTGACATCCGTAGAGTGGCTATCGACCTCGTACGCTCTCCTACTGGTATGCAGTCTGGTGCCTCAGGTAAGTTCGGTGCAACAGCTTCTGAc                                                                                                                 <  1:129654/292‑1 (MQ=255)`  
                                                                                                                                                                                            `tGAACATAATGGTGTCCCTTATGAGGACTTACCAACAGAGAGAGGTGCCGTGGTGTTACATGATGGCTCCGTTCTAAGAGCAAGCAACCCAATCAACCCTAAGACTCTAAACGAGTTCTCCGAGGTTGACCCTGAGAAGGCTGCGCGAGGAATAAAACTGGCT‑GGGTTCACCGAGATTGGCTTGAAGACCTTGGGGTCTGACGATGCTGACATCCGTAGAGTGGCTATCGACCTCGTTCGCTCTCCTACTGGTATGCAGTCTGGTGCCTCAGGTAAGTTCGGTGCAACAGCTTCTGACATc                                                                                                              <  1:285524/301‑1 (MQ=255)`  
                                                                                                                                                                                             `gAACATAATGGTGTCCCTTATGAGGACTTACCAACAGAGAGAGGTGCCGTGGTGTTACATGATGGCTCCGTTCTAAGTGCAAGCAACCCAATCAACCCTAAGACTCTAAACGAGTTCTCCGAGGTTGACCCTGAGAAGGCTGCGCGAGGAATCAAACTGGCT‑GGGTTCACCGAGATTGGCTTGAAGACCTTGGGGTCTGACGATGCTGACATCCGTAGAGTGGCTATCGACCTCGTTCGCTCTCCTACTGGTATGCAGTCTGGTGCCTCAGGTAAGTTCGGTGCAACAGCTTCTGACATcc                                                                                                             >  1:127066/1‑301 (MQ=255)`  
                                                                                                                                                                                               `aCATAATGGTGTCCCTTATGAGGACTTACCAACAGAGAGAGGTGCCGTGGTGTTACATGATGGCTCCGTTCTAAGTGCAAGCAACCCAATCAACCCTAAGACTCTAAACGAGTTCTCCGAGGTTGACCCTGAGAAGGCTGCGCGAGGAATCAAACTGGCT‑GGGTTCACCGAGATTGGCTTGAAGACCTTGTGGTCTGACGATGCTGACATCCGTAGAGTGGCTATCGACCTCGTTCGCTCTCCTACTGGTATGCAGTCTGGTGCCTCAGGTAAGTTCGGTGcctgtctcttatacacatct                                                                                                           >  1:192052/1‑282 (MQ=255)`  
                                                                                                                                                                                                  `gtataagagacagtATGAGGACTTACCAACAGAGAGAGGTGCCGTGGTGTTACATGATGGCTCCGTTCTAAGTGCAAGCAACCCAATCAACCCTAGGACTCTAAACGAGTTCTCCGAGGTTGACCCTGAGAAGGCTGCGCGAGGAATCAAACTGGCT‑GGGTTCACCGAGATTGGCTTGAAGACCTTGGGGTCTGACGATGCTGACATCCGTAGAGTGGCTATCGACCTCGTTCGCTCTCCTACTGGTATGCAGTCTGGTGCCTCAGGTAAGTTCGGTGCAACAGCTTCTGACATCCATgag                                                                                                        <  1:143119/288‑1 (MQ=255)`  
                                                                                                                                                                                                    `aTGGTGTCCCTTATGAGGACTTACCAACAGAGAGAGGTGCCGTGGTGTTACATGATGGCTCCGTTCTAAGTGCAAGCAACCCAATCAACCCTAAGACTCTAAACGAGTTCTCCGAGGTTGACCCTGAGAAGGCTGCGCGAGGAATCAAACTGGCT‑GGGTTCACCGAGATTGGATTGAAGACCTTGGGGTCTGACGATGCTGACATCCGTAGAGTGGCTATCGACCTCGTTCGCTCTCCTACTGGTATGCAGTCTGGTGCCTCAGGTAAGTTCGGTGCAACAGCTTCTGACATCCATgagag                                                                                                      >  1:195238/1‑301 (MQ=255)`  
                                                                                                                                                                                                    `aTGGGGTACCGTATGAGGACTTAGCAACAGAGAGAGGTGCCGTAGTGTTACATGATGGCTCCGTTCTAAGTGCAAGCAACCCAATCAACCCTAAGACTCTAAAAGAGTTCTCCGAGGTTGACCCTGAGAAGGCTGCGCGAGGAATCAAACTGGCT‑GGGTTCACCGAGATTGGCTTGAAGACCTTGGGGTCTGACGATGCTGACATCCGTAGAGTGGCTATCGACCTCGTTCGCTCTCCTACTGGTATGCAGTATGGTGCCTCAGGTAAGTTCGGTGCAACAGCTTCTGACATCCATgagag                                                                                                      <  1:27290/301‑1 (MQ=255)`  
                                                                                                                                                                                                      `ggTGTCCCTTATGAGGACTTACCAACAGAGAGAGGTGCCGTGGTGTTACATGATGGCTCCGTTCTAAGCGCAAGCAACCCAATCAACCCTAAGACTCTAAAAGAGTTCTCCGAGGTTGACCCTGAGAAGGCTGCGCGAGGAATCAAACTGGCT‑GGGTTCACCGAGATTGGCTTGAAGACCTTGGGGTCTGACGATGCTGACATCCGTAGAGTGGCTATCGACCTCGTTCGCTCTCCTACTGGTATGCAGTCTGGTGCCTCAGGTAAGTTCGGTGCAACAGCTTCTGACATCCATGAGAGAc                                                                                                    >  1:94891/1‑301 (MQ=255)`  
                                                                                                                                                                                                           `cccTTATGAGGACTTACCAACAGAGAGAGGTGCCGTGGTGTTACATGATGGCTCCGTTCTAAGTGCAAGCAACCCAATCAACCCTAAGACTCTAAAAGAGTTCTCCGAGGTTGACCCTGAGAAGGCTGCGCGAGGAATCAAACTGGCT‑GGGTTCACCGAGATTGGCTTGAAGACCTTGGGGTCTGACGATGCTGACATCCGTAGAGTGGCTATCGACCTCGTTCGCTCTCCTACTGGTATGCAGTCTGGTGCCTCAGGTAAGTTCGGTGCAACAGCTTCTGACATCCATGAGAGACTTCAt                                                                                               >  1:268089/1‑301 (MQ=255)`  
                                                                                                                                                                                                              `ttATGAGGACTTACCAACAGAGAGAGGTGCCGTGGTGTTACATGATGGCTCCGTTCTAAGTGCAAGCAACCCAATCAACCCTAAGACTCTAAACGAGTTCTCCGAGGTTGACCCTGAGAAGGCTGCGCGAGGAATCAAACTGGCT‑GGGTTCACCGAGATTGGCTTGAAGACCTTGGGGTCTGACGATGCTGACATCCGTAGAGTGGCTATCGACCTCGTTCGCTCTCCTACTGGTATGCAGTCTGGTGCCTCAGGTAAGTTCGGTGCAACAGCTTCTGACATCCATGAGAGACTTCATGGt                                                                                            <  1:311516/301‑1 (MQ=255)`  
                                                                                                                                                                                                               `tATGAGGACTTACCAACAGAGAGAGGTGCCGTGGTGTTACATGATGGCTCCGTTCTAAGTGCAAGCAACCCAATCAACCCTAAGACTCTAAAAGAGTTCTCCGAGGTTGACCCTGAGAAGGCTGCGCGAGGAATCAAACTGGCT‑GGGTTCACCGAGATTGGCTTGAAGACCTTGGGGTCTGACGATGCTGACATCCGTAGAGTGGCTATCGACCTAGTTCGCTCTCCTACTGGTATGCAGTCTGGTGCCTCAGGTAAGTTCGGTGCAACAGCTTCTGACATCCATGAGAGACTTCATGGTa                                                                                           >  1:12472/1‑301 (MQ=255)`  
                                                                                                                                                                                                                `aTGAGGTCTTACCAACAGAGAGAGGTGCCGTGGTGTTACATGATGGCTCCGTGCTAAGTGCAAGCAACCCAATCAACCCTAAGACTCTAAACGAGTTCTCCGAGGTTGACCCTGAGAAGGCTGCGCGATGAATCAAACTGGCT‑GGGTTCACCGAGATTGGCTTGAAGACCTTGGGGTCTGACGATGCTGACATCCGTAGAGTGGCTATCGACCTCGTTCGCTCTCCTACTGGTATGCAGTCTGGTGCCTCAGGTAAGTTCGGTGCAACAGCTTCTGACATCCATGAGAGACTTCATGGTAc                                                                                          <  1:41224/301‑1 (MQ=255)`  
                                                                                                                                                                                                                    `ggACTTACCAACAGAGAGAGGTGCCGTGGTGTTACATGATGGCTCCGTTCTAAGTGCAAGCAACCCAATCAACCCTAAGACTCTAAACGAGTTCTCCGAGGTTGACCCTGAGAAGGCTGCGCGAGGAATCAAACTGGCT‑GGGTTCACCGCGATTGGCTTGAAGACCTTGGGGTCTGACGATGCTGACATCCGTAGAGTGGCTATCGACCTCGTTCGCTCTCCTACTGGTATGCAGTCTGGTGCCTCAGGTAAGTTCGGTGCAACAGCTTCTGACATCCATGAGAGACTTCATGGTACTGAc                                                                                      >  1:152611/1‑301 (MQ=255)`  
                                                                                                                                                                                                                    `ggACTTACCAACAGAGAGAGGTGCCGTGGTGTTACATGATGGCTCCGTTCTAAGTGCAAGCAACCCAATCAACCCTAAGACTCTAAACGAGTTCTCCGAGGTTGACCCTGAGAAGGCTGCGCGAGGAATCAAACTGGCT‑GGGTTCACCGAGATTGGCTTGAAGACCTTGGGGTCTGACGATGCTGACATCCGTAGAGTGGCTATCGACCTCGTTCGCTCTCCTACTGGTATGCAGTCTGGTGCCTCAGGTAAGTTCGGTGCAACAGCTTCTGACATCCATGAGAGACTTCATGGTACTGAc                                                                                      >  1:78889/1‑301 (MQ=255)`  
                                                                                                                                                                                                                     `gACTTACCAACATGTAGAGGCTCCTTGTTTTTTCCTGATGGCTCCGTTCTAAGTGCAAGCAACCCAATCAACCCTAATACTCTAAACGAGTTCTCCGAGGTTGACCCTTAGAAGGCTGCGCGAGGAATCAATCTGGCT‑GGGTTCACCGGTATTGGCTTGCAGACCTTGGGGTCTGACGATGCTGACATCCGTAGAGTGGCTATCGATCTCGTTCGCTCTCCTACTGGTATGCAGTCTGGTGCCTCAGGTAAGTTCGGTGCAACAGCTTCTGACATCCATGAGAGACTTCATGGTACTGAcc                                                                                     <  1:219965/301‑1 (MQ=255)`  
                                                                                                                                                                                                                     `gACTTACCAACAGAGAGAGGTGCCGTGGTGTTACATGATGGCTCCGTTCTAAGTGCAAGCAACCCAATCAACCCTAAGACTCTAAACGAGTTCTCCGAGGTTGACCCTGAGAAGGCTGCGCGAGGAATCAAACTGGCT‑GGGTTCACCGAGATTGGCTTGAAGACCTTGGGGTCTGACGATGCTGACATCCGTAGAGTGGCTATCGACCTCGTTCGCTCTCCTACTGGTATGCAGTCTGGTGCCTCAGGTAAGTTCGGTGCAACAGCTTCTGACATCCATGAGAGACTTCATGGTACTGAcc                                                                                     <  1:8508/301‑1 (MQ=255)`  
                                                                                                                                                                                                                     `gACTTACCAAAAGAGAGAGGTGCCGTGGTGTTACAAGATGGCGCCGTTCTAAGTACAAGCAACCCAATCAACCCTAAGACTCTAAAAGAGTTCTCCGAGGTTGACCCTGAGAAGGCTGCGCGAGGAATCAAACTGGCT‑GGGTTCACCGAGATTGGCTTGAAGACCTTGGGGTCTGACGATGCTGACATCCGTAGAGTGGCTATCGACCTCGTTCGCTCTCCTACTGGTATGCAGTCTGGTGCCTCAGGTAAGTTCGGTGCAACAGCTTCTGACATCCATGAGAGACTTCATGGTACTGAcc                                                                                     <  1:292840/301‑1 (MQ=255)`  
                                                                                                                                                                                                                     `agcTTACCAACAGAGAGAGGAGCCGTGGTGTTACATGATGGCTCCGTTCTAAGTGCAAGCAACCCAATCAACCCTAAGACTCTAAAAGAGTTCTCCGAGGTTGACCCTGAGAAGGCTGCGCGAGGAATCAAACTGGCT‑GGGTTCACCGAGATTGGCTTGAAGACCTTGGGGTCTGACGATGCTGACATCCGTAGAGTGGCTATCGACCTCGTTCGCTCTCCTACTGGTATGCAGTCTGGTGCCTCAGGTAAGTTCGGTGCAACAGCTTCTGACATCCATGAGAGACTTCATGGTACTGAcc                                                                                     <  1:314968/299‑1 (MQ=255)`  
                                                                                                                                                                                                                      `aCTTACCAACAGAGAGAGGTGCCGTGGTGTTACATGATGGCTCCGTTCTAAGTGCAAGCAACCCAATCAACCCTAAGACTCTAAACGATTTCTCCGAGGTTTACCCTGAGAAGTCTGCGCGAGGAATCAAACTTGCT‑GGGTTCACCGAGATTGGCTTGAAGACCTTGGGGTCTGACGATGCTGACATCCGTAGAGTGGCTATCGACCTCGTTCGCACTCCTACTGGTTTGCAGTCTGGTGACTCAGGTCAGTTCGGTGCACCAGCTTCTGCCATCCATGCTCGACTTCTTGGTACTGAccg                                                                                    >  1:213451/1‑300 (MQ=255)`  
                                                                                                                                                                                                                       `cTTACCAACAGAGAGAGGTGCCGTGGTGTTACATGATGGCTCCGTTCTAAGTGCAAGCAACCCAATCAACCCTAAGACTCTAAACGAGTTCTCCGAGGTTGACCCTGAGAAGGCTGCGCGAGGAATCAAACTGGCT‑GGGTTCACCGAGATTGGCTTGAAGACCTTGGGGTCTGACGATGCTGACATCCGTAGAGTGGCTATCGACCTCGTTCGCTCTCCTACTGGTATGCAGTCTGGTGCCTCAGGTAAGTTCGGTGCAACAGCTTCTGACATCCATGAGAGACTTCATGGTACTGACCAg                                                                                   >  1:288413/1‑301 (MQ=255)`  
                                                                                                                                                                                                                          `acagaCAGAGAGAGGTGCCGTGGTGTTACATGATGGCTCCGTTCTAAGTGCAAGCAACCCAATCAACCCTAAGACTCTAAACGAGTTCTCCGAGGTTGACCCTGAGAAGGCTGCGCGAGGAATCAAACTGGCT‑GGGTTCACCGAGATTGGCTTGAAGACCTTGGGGTCTGACGATGCTGACATCCGTAGAGTGGCTATCGACCTCGTTCGCTCTCCTACTGGTATGCAGTCTGGTGCCTCAGGTAAGTTCGGTGCAACAGCTTCTGACATCCATGAGAGACTTCATGGTACTGACCAGCGt                                                                                <  1:103157/297‑1 (MQ=255)`  
                                                                                                                                                                                                                               `cAGAGAGAGGTGCCGTGGTGTTACATGATGGCTCCGTTCTAAGTGCAAGCAACCCAATCAACCCTAAGGCGCTAAACGAGTTCTCCGAGGTTGACCCTGAGAAGGCTGCGCGAGGAATCAAACTGGCT‑GGGTTCACCGAGATTGGCTTGAAGACCTTGGGGTCTGACGATGCTGACATCCGTAGAGTGGCTATCGACCTCGTTCGCTCTCCTACTGGTATGCAGTCTGGTGCCTCAGGTAAGTTCGGTGCAACAGCTTCTGACATCCATGAGAGACTTCATGGTACTGACCAGCGTACTta                                                                           <  1:51001/301‑1 (MQ=255)`  
                                                                                                                                                                                                                               `cAGAGAGAGGTGCCGTGGTGTTACATGATGGCTCCGTTCTAAGTGCAAGCAACCCAATCAACCCTAAGACTCTAAACGAGTTCTCCGAGGTTGACCCTGAGAGGGCTGCGCGAGGAATCAAACTGGCT‑GGGTTCACCGAGATTGGCTTGAAGAACGTGGGGTCTGACGATGCTGACATCCGTAGAGTGGCTATCGACCTCGTTCGCTCTCCTACAGGTATTCAGTCTGGTGCCTCAGGTAACTTCGGTTCAACAGCTTCTTGAATCCATGCAGGACTTCATGATACTGACCAGCGGACCta                                                                           >  1:305576/1‑301 (MQ=255)`  
                                                                                                                                                                                                                                    `tataagagacaggtgTTACATGATGGCTCCGTTCTAAGTGCAAGCAACCCAATCAACCCTAAGACTCTAAACGAGTTCTCCGAGGTTGACCCTGAGAAGGCTGCGCGAGGAATCAAACTGGCT‑GGGTTCACCGAGATTGGCTTGAAGACCTTGGGGTCTGACGATGCTGACATCTGTAGAGTGGCTATCGACCTCGTTCGCTCTCCTACTGGTATGCAGTCTGGTGCCTCAGGTAAGTTCGGTGCAACAGCTTCTGACATCCATGAGAGACTTCATGGTACTGACCAGCGTACTTATAATg                                                                      <  1:119474/290‑1 (MQ=255)`  
                                                                                                                                                                                                                                    `accgttGCCGTTGTATTACATTATTCTTCCGTTCTACCTTCAAGCCACCCATTCAACCCTTAGACTCTACTCTAGTTCTCCGAGGTTTACCCTGAGAAGGCTGCGCGAGGATTCCCACTGTCT‑GGTTTCCCCGAGATTGGCTTGTAGACCTTGGGATCTGACGATTCTTCCATCCGTAGAGTGGCTATCGACCTCGTTCGCTCTCCTACTGATATGCAGTCTGGTGCCTCAGGTAAGTTCGGTGCAACAGCTTCTGACATCCATGAGAGACTTCATGGTACTGACCAGCGTACTTATAATg                                                                      <  1:179551/296‑1 (MQ=255)`  
                                                                                                                                                                                                                                     `gagGTGCCGTGGTGTTACATGATGGCTCCGTTCTAAGTGCAAGCAACCCAATCAACCCTAAGACTCTAAACGAGTTCTCCGAGGTTGACCCTGAGAAGGCTGCGCGAGGAATCAAACTGGCT‑GGGTTCACCGAGATTGGCTTGAAGACCTTGGGGTCTGACGATGCTGACATCCGTAGAGTGGCGATCGACCTCGTTCGCTCTCCTACTGGTATGCAGTCTGGTGCCTCAGGTAAGTTCGGTGCAACAGCTTCTGACATCCATGAGAGACTTCATGGTACTGTCCAGCGTACTTATAATGa                                                                     >  1:229599/1‑301 (MQ=255)`  
                                                                                                                                                                                                                                       `ggTGCCGTGGTGTTACATGATGGCTCCGTTCTAAGTGCAAGCAACTCAATCAACCCTAAGACTCTAAACGAGTTCTCCGAGGTTGACCCTGAGAAGGCTGCGCGAGGAATCAAACTGGCT‑GGGTTCACCGAGATTGGCTTGAAGACCTTGGGGTCTGACGATGCTGACATCCGTAGAGTGGCTATCGACCTCGTTCGCTCTCCTACTGGTATGCAGTCTGGTGCCTCAGGTAAGTTCGGTGCAACAGCTTCTGACATCCATGAGAGACTTCATGGTACTGACCAGCGTACTTATAATGACt                                                                   >  1:18763/1‑301 (MQ=255)`  
                                                                                                                                                                                                                                       `ggTGCCGTGGTGTTACATGATGGCTCCGTTCTAAGTGCAAGCAACCCAATCAACCCTAAGACTCTAAACGAGTTCTCCGAGGTTGACCCTGAGAAGGCTGCGCGAGGAATCAAACTGGCT‑GGGTTCACCGAGATTGGCTTGAAGACCTTGGGGTCTGACGATGCTGACATCCGTAGAGTGGCTATCGACCTCGTTCGCTCTCCTACTGGTATGCAGTCTGGTGCCTCAGGTAAGTTCGGTGCAACAGCTTCTGACATCCATGAGAGACTTCATGGTACTGACCAGCGTACTTATAATGACt                                                                   <  1:110547/301‑1 (MQ=255)`  
                                                                                                                                                                                                                                       `ggTGCCGTGGTGTTACATGATGGCTCCGTTCTAAGTGCAAGCAACCCAATCAACCCTAAGACTCTAAACGAGTTCTCCGAGGTTGACCATGAGAAGGCTGCGCGAGGAATCAAACTGGCT‑GGGTTCACCGAGATTGGCTTGAAGACCTTGGGGTCTGACGATGCTGACATCCGTAGAGTGGCTATCGACCTCGTTCGCTCTCCTACTGGTATGCAGTCTGGTGCCTCAGGTAAGTTCGGTGCAACAGCTTCTGACATCCATGAGAGACTTCATGGTACTGACCAGCGTACTTATAATGACt                                                                   <  1:227178/301‑1 (MQ=255)`  
                                                                                                                                                                                                                                       `ggTGCCGTGGTGTTACATGATGGCTCCGTTCTAAGTGCAAGCAACCCAATCAACCCTAAGACTCTAAAAGAGTTCTCCGAGGTTGACCCTGAGAAGGCTGCGCGAGGAATCAAACTGGCT‑GGGTTCACCGAGATTGGCTTGAAGACCTTGGGGTCTGACGATGCTGACATCCGTAGAGTGGCTATCGACCTCGTTCGCTCTCCTACTGGTATGCAGTCTGGTGCCTCGGGTAAGTTCGGTGCAACAGCTTCTGACATCCATGAGAGACTTCATGGTACTGACCAGCGTACTTATAATgctt                                                                   >  1:59081/1‑298 (MQ=255)`  
                                                                                                                                                                                                                                       `ggTGCCGTGGTGTTACATGATGGCTCCGTTCTAAGTGCAAGCAACCCAATCAACCCTAAGACTCTAAAAGAGTTCTCCGAGGTTGACCCTGAGAAGGCTGCGCGAGGAATCAAACTGGCT‑GGGTTCACCGAGATTGGCTTGAAGACCTTGGGGTCTGACGATGCTGACATCCGTAGAGTGGCTATCGACCTCGTTCGCTCTCCTACTGGTATGCAGTCTGGTGCCTCAGGTAAGTTCGGTGCAACAGCTTCTGACATCCATGAGAGACTTCATGGTACTGACCAGCGTACTTATAATGACt                                                                   <  1:209811/301‑1 (MQ=255)`  
                                                                                                                                                                                                                                       `gctcggagatgtgtataagagacagccGTTCTAAGTGCAAGCAACCCAATCAACCCTAAGACTCTAAACGAGTTCTCCGAGGTTGAACCTGAGAAGGCTGCGCGAGGAATCAAACTGGCT‑GGGTTCACCGAGATTGGCTTGAAGACCTTGGGGTCTGACGATGCTGACATCCGTAGAGTGGCTATCGACCTCGTTCGCTCTCCTACTGGTATGCAGTCTGGTGCCTCAGGTAAGTTCGGTGCAACAGCTTCTGACATCCATGAGAGACTTCATGGTACTGACCAGCGTACTTATAATGACt                                                                   <  1:164629/276‑1 (MQ=255)`  
                                                                                                                                                                                                                                          `gCCGTGGTGTTACATGATGGCTCCGTTCTAAGTGCAAGCAACCCAATCAACCCTAAGACTCTAAACGAGTTCTCCGAGGTTGACCCTGAGAAGGCTGCGCGAGGAATCAAACTGGCT‑GGGTTCACCGAGATTGGCTTGAAGACCTTGGGGTCTGACGATGCTGACATCCGTAGAGTGGCTATCGACCTCGTTCGCTCTCCTACTGGTATGCAGTCTGGTGCCTCAGGTAAGTTCGGTGCAACAGCTTCTGACATCCATGAGAGCCTTCATGGTACTGACCAGCGTACTTATAATGACTTGt                                                                >  1:337265/1‑301 (MQ=255)`  
                                                                                                                                                                                                                                            `cGTGGTGTTAAATGATGGCTCCGTTCTAAGTGCAAGCAACCCAATAAACCCTAAGACTCTAAAAGAGTTCTCCGAGGTTGACCCTGAGAAGGCTGCGCGAGGAATCAAACTGGCT‑GGGTTCACCGAGATTGGCTTGAAGACCTTGGGGTCTGACGATGCTGACATCCGTAGAGTGGCTATCGACCTCGTTCGCTCTTCTACTGGTATGCAGTCTGGTGCCTCAGGTAAGTTCGGTGCAACAGCTTCTGACATCCATGAGAGACTTCATGGTACTGACCAGCGTACTTATAATGACTTGTAc                                                              <  1:74948/301‑1 (MQ=255)`  
                                                                                                                                                                                                                                                `gtgtTACATGATGGCTCCGTTCTAAGTGCAAGCAACCCAATCAACCCTAAGACTCTAAACGAGTTCTCCGAGGTTGACCCTGAGAAGGCTGCGCGAGGAATCAAACTGGCT‑GGGTTCACCGAGATTGGCTTGAAGACCTTGGGGTCTGACGATGCTGACATCCGTAGAGTGGCTATCGACCTCGTTCGCTCTCCTACTGGTATGCAGTCTGGTGCCTCAGGTAAGTTCGGTGCAACAGCTTCTGACATCCATGAGAGACTTCATGGTACTGACCAGCGTACTTATAATGACTTGTACAAAg                                                          >  1:299066/1‑301 (MQ=255)`  
                                                                                                                                                                                                                                                `gtgtTACATGATGGCTCCGTTCTAAGTGCAAGCAACCCAATCAACCCTAAGACTCTAAACGAGTTCTCCGAGGTTGACCCTGAGAAGGCTGCGCGAGGAATCAAACTGGCT‑GGGTTCACCGAGATTGGCTTGAAGACCTTGGGGTCTGACGATGCTGACATCCGTAGAGTGGCTATCGACCTCGTTCGCTCTCCTACTGGTATGCAGTCTGGTGCCTCAGGTAAGTTCGGTGCAACAGCTTCTGACATCCATGAGAGACTTCATGGTACTGACCAGCGTACTTATAATGACTTGTACAAAg                                                          >  1:31868/1‑301 (MQ=255)`  
                                                                                                                                                                                                                                                `gtgtTACATGATGGCTCCGTTCTAAGTGCAAGCAACCCAATCAACCCTAAGACTCTAAACGAGTTCTCCGAGGTTGACCCTGAGAAGGCTGCGCGAGGAATCAAACTGGCT‑GGGTTCACCGAGATTGGCTTGAAGACCTTGGGGTCTGACGATGCTGACATCCGTAGAGTGGCTATCGACCTCGTTCGCTCTCCTACTGGTATGCAGTCTGGTGCCTCAGGTAAGTTCGGTGCAACAGCTTCTGACATCCATGAGAGACTTCATGGTACTGACCAGCGTACTTATAATGACTTGTACAAAg                                                          >  1:262175/1‑301 (MQ=255)`  
                                                                                                                                                                                                                                                `gtgtTACATGATGGCTCCGTTCTAAGTGCAAGCAACCCAATCAACCCTAAGACTCTAAACGAGTTCTCCGAGGTTGACCCTGAGAAGGCTGCGCGAGGAATCAAACTGGCT‑GGGTTCACCGAGATTGGCTTGAAGACCTTGGGGTCTGACGATGCTGACATCCGTAGAGTGGCTATCGACCTCGTTCGCTCTCCTACTGGTATGCAGTCTGGTGCCTCAGGTAAGTTAGGTGCAACAGCTTCTGACATCCATGAGAGACTTCATGGTACTGACCAGCGTACTTATAATGATTTGTACAAAg                                                          >  1:347718/1‑301 (MQ=255)`  
                                                                                                                                                                                                                                                 `tgtTACATGATGGCTCCGTTCTAAGTGCAAGCAACCCAATCAACCCTAAGACTCTAAACGAGTTCTCCGAGGTTGACCCTGATAAGGCTGCGCGAGGAATCAAACTGGCT‑GGGTTCACCGAGATTGGCTTGAAGACCTTGGGGTCTGACGATGCTGACATCCGTAGAGTGGCTATCGACCTCGTTCGCTCTCCTACTGGTATGCAGTCTGGTGCCTCAGGTAAGTTCGGTGCAACAGCTTCTGACATCCATGAGAGACTTCATGGTACTGACCAGCGTACTTATAATGACTTGTACAAAGc                                                         <  1:283261/301‑1 (MQ=255)`  
                                                                                                                                                                                                                                                 `tgtTACATGATAGCTCCGTTCTAAGTGCAAGCAACCCAATCAACCCTAAGACTCTAAAAGAGTTCTCCTAGGTTGACCCTGAGAAGGCTGCGCGAGGAATCAAACTGGCT‑GGGTTCACCGAGATTGGCTTGAAGACCTTGGTGTCTGACGATGCTGACATCCGTAGAGTGGCTATCGACCTCGTTCGCTCTCCTACTGGTATGCAGTCTGGTGCCTCAGGTAAGTTCGGTGCAACAGCTTCTGACATCCATGAGAGACTTCATGGTACTGACCAGCGTACTTATAATGACTTGTACAAAGc                                                         <  1:135506/301‑1 (MQ=255)`  
                                                                                                                                                                                                                                                 `ggtTACATGATGGCTCCGTTCTAAGTGCAAGCAACCCAATCAACCCTAAGACTCTAAACGAGTTCTCCGAGGTTGACCCTGAGAAGGCTGCGCGAGGAATCAAACTGGCT‑GGGTTCACCGAGATTGGCTTGAAGACCTTGGGGTCTGACGATGCTGACATCCGTAGAGTGGCTATCGACCTCGTTCGCTCTCCTACTGGTATGCAGTCTGGTGCCTCAGGTAAGTTCGGTGCAACAGCTTCTGACATCCATGAGAGACTTCATGGTACTGACCAGCGTACTTATAATGACTTGTACAAAGc                                                         <  1:27084/300‑1 (MQ=255)`  
                                                                                                                                                                                                                                                 `gagacagtgatgGCTCCGTTCTAAGTGCAAGCAACCCAATCAACCCTAAGACTCTAAACGAGTTCTCCGAGGTTGACCCTGAGAAGGCTGCGCGAGGAATCAAACTGGCT‑GGGTTCACCGAGATTGGCTTGAAGACCTTGGGGTCTGACGATGCTGACATCCGTAGAGTGGCTATCGACCTCGTTCGCTCTCCTACTGGTATGCAGTCTGGTGCCTCAGGTAAGTTCGGTGCAACAGCTTCTGACATCCATGAGAGACTTCATGGTACTGACCAGCGTACTTATAATGACTTGTACAAAGc                                                         <  1:100069/294‑1 (MQ=255)`  
                                                                                                                                                                                                                                                      `cATGATGGCTCCGTTCTAAGTGCAAGCAACCCAATCAACCCTAAGACTCTAAAAGAGTTCTCCGAGGTTGACCCTGAGAAGGCTGCGCGAGGAATCAAACTGGCT‑GGGTTCACCGAGATTGGCTTGAAGACCTTGGGGTCTGACGATGCTGACATCCGTAGAGTGGCTATCGACCTCGTTCGCTCTCCTACTGGTATGCAGTCTGGTGCCTCAGGTAAGTTCGGTGCATCAGCTTCTGACATCCATGAGAGACTTCATGGTACTGACCAGCGTACTTATAATGACTTGTACAAAGCAATGt                                                    >  1:33488/1‑301 (MQ=255)`  
                                                                                                                                                                                                                                                             `gCTCCGTTCTAAGTGCAAGCAACCCAATCAACCCTAAGACTCTAAACGAGTTCTCCGAGGTTGACCCTGAGAAGGCTGCGCGAGGAATCAAACTGGCT‑GGGTTCACCGAGATTGGCTTGAAGACCTTGGGGTCTGACGATGCTGACATCCGTAGAGTGGCTATCGACCTCGTTCGCTCTCCTACTGGTATGCAGTCTGGTGCCTCAGGTAAGTTCGGTGCAACAGCTTCTGACATCCATGAGAGACTTCATGGTACTGACCAGCGTACTTATAATGACTTGTACAAAGCAATGTCTGACGc                                             >  1:308249/1‑301 (MQ=255)`  
                                                                                                                                                                                                                                                                `ccGTTCTAAGTGCAAGCAACCCAATCAACCCTAAGACTCTAAACGAGTT‑TCCGAGGTTGACCCTGAGAAGGCTGCGCGAGGAATCAAACTGGCT‑GGGTTCACCGAGATTGGCTTGAAGACCTTGGGGTCTGACGATGCTGACATCCGTAGAGTGGCTATCGACCTCGTTCGCTCTCCTACTGGTATGCAGTCTGGTGCCTCAGGTAAGTTCGGTGCAACAGCTTCTGACATCCATGAGAGACTTCATGGTACTGACCAGCGTACTTATAATGACTTGTACAAAGCAATGTCTGACGCTAtt                                         >  1:223637/1‑300 (MQ=255)`  
                                                                                                                                                                                                                                                                  `gTTCT‑AGTGCAAGCAACCCAATCAACCCTAAGACTCTAAACGAGTTCTCCGAGGTTGACCCTGAGAAGGCTGCGCGAGGAATCAAACTGGCT‑GGGTTCACCGAGATTGGCTTGAAGACCTTGGGGTCTGACGATGCTGACATCCGTAGAGTGGCTATCGACCTCGTTCGCTCTCCTACTGGTATGCAGTCTGGTGCCTCAGGTAAGTTCGGTGCAACAGCTTCTGACATACATGAGAGACTTCATGGTACTGACCAGCGTACTTATAATGACTTGTACAAAGCACTGTCTGACGCTATGaa                                       >  1:67499/1‑301 (MQ=255)`  
                                                                                                                                                                                                                                                                     `tcggagatgtgtataagagacagcccTAAGACTCTAAAAGAGTTCTCCGAGGTTGACCCTGAGAAGGCTGCGCGAGGAATCAAACTGGCT‑GGGTTCACCGAGATTGGCTTGAAGACCTTGGGGTCTGACGATGCTGACATCCGTAGAGTGGCTATCGACCTCGTTCGCTCTCCTACTGGTATGCAGTCTGGTGCCTCAGGTAAGTTCGGTGCAACAGCTTCTGACATCCATGAGAGACTTCATGGTACTGACCAGCGTACTTATAATGACTTGTACAAAGCAATGTCTGACGCTATGAAAg                                     <  1:171578/278‑1 (MQ=255)`  
                                                                                                                                                                                                                                                                       `aaGTGCAAGCAACCCAATCAACCCTAAGACTCTAAACGAGTTCTCCGAGGTTGACCCTGAGAAGGCTGCGCGAGGAATCAAACTGGCT‑GGGTTCACCGATATGGGCTTGAAGACCTTGGGGTCTGACGATGCTGACATCCGTAGAGTGTCTATCGATCTCGTTCGCTCTCCTACTGGTATGCAGTCTGGTGCCTCAGGTAAGTTCGGTGCAACAGCTTCTGACATCCATGAGAGACTTCATGGTACTGACCAGCGTACTTATAATGACTTGTACAAAGCAATGTCTGACGCTATGAAAGAc                                   <  1:121809/301‑1 (MQ=255)`  
                                                                                                                                                                                                                                                                        `aGTGCAAGCAACCCAATCAACCCTAAGACTCTAAACGAGTTCTCCGAGGTTGACCCTGAGAAGGCTGCGCGAGGAATCAAACTGGCT‑GGGTTCACCGAGATTGGCTTGAAGACCTTGGGGTCTGACGATGCTGACATCCGTAGAGTGGCTATCGACCTCGGTCGCTCTCCTACTGGTATGCAGTCTGGTGCCTCAGGTAAGTTCGGTGCAACAGCTTCCGACATCCATGAGAGACTTCATGGTACTGACCAGCGTACTTATAATGACTTGTACAAAGAAATGTCTGACGCTATGAAAGAcc                                  >  1:332221/1‑301 (MQ=255)`  
                                                                                                                                                                                                                                                                                `caaCCCAATCAACCCTAAGACTCTAAAAGAGTTCTCCGAGGTTGACCCTGAGAAGGCTGCGCGAGGAATCAAACTGGCT‑GGGTTCACCGAGATTGGCTTGAAGACCTTGGGGTCTGACGATGCTGACATCCGTAGAGTGGCTATCGACCTCGTTCGCTCTCCTACTGGTATGCAGTCTGGTGCCTCAGGTAAGTTCGGTGCAACAGCTTCTGACATCCATGAGAGACTTCATGGTACTGACCAGCGTACTTATAATGACTTGTACAAAGCAATGTCTGACGCTATGaacggtcgcttatac                          >  1:324441/1‑288 (MQ=255)`  
                                                                                                                                                                                                                                                                                   `cccAATCGACCCTAAGACTCTAAAAGAGTTCTCCGAGGTTGACCCTGAGAAGGCTGCGCTAGGAATCAAACGGGCT‑GGGTTCACCGAGATTGGCTTGAAGACCTTGGGGTCTGACGATGCTGACATCCGTAGAGTGGCTATCGACCTCGTTCGCTCTCCTACTGGTATGCAGTCTGGTGCCTCAGGTAAGTTCGGTGCAACAGCTTCTGACATCCATGAGAGACTGCATGGTACTGACCAGCGTACTTATAATGACTTGTACAAAGCAATGTCTGACGCTATGAAAGACCCTGAGTTctct                       <  1:253326/301‑1 (MQ=255)`  
                                                                                                                                                                                                                                                                                   `cccAATCAACCCTAAGACTCTAAACGAGTTCTCCGAGGTTGACCCTGAGAAGGCTGCGCGAGGAATCAAACTGGCT‑GGGTTCACCGAGATTGGCTTGAAGACCTTGGGGTCTGACGATGCTGACATCCGTAGAGTGGCTATTGACCTCGTTCGCTCTCCTACTGGTATGCAGTCTGGTGCCTCAGGTAAGTTCGGTGCAACAGCTTCTGACATCCATGAGAGACTTCATGGTACTGACCAGCGTACTTATAATGACTTGTACAAAGCAATGTCTGACGCTATGAAAGACCCTGAGTTctct                       <  1:279459/301‑1 (MQ=255)`  
                                                                                                                                                                                                                                                                                    `ccAATCAACCCTAAGACTCTAAAAGAGTTCTCCGAGGTTGACCCTGAGAAGGCTGCGCGAGGAATCAAACTGGCT‑GGGTTCACCGAGATTGGCTTGAAGACCTTGGGGTCTGACGATGCTGACATCCGTAGAGTGGCTATCGACCTCGTTCGCTCTCCTACTGGTATGCAGTCTGGTGCCTCAGGTAAGTTCGGTGCAACAGCTTCTGACATCCATGAGAGACTTCATGGTACTGACCAGCGTACTTATAATGACTTGTACAAAGCAATGTCTGACGCTATGAAAGACCCTGAGTTCTCTa                      >  1:16674/1‑301 (MQ=255)`  
                                                                                                                                                                                                                                                                                     `cAATCAACCATAAGACTCTAAACGAGTTCGCCGAGGTTGACCATGAGAAGGCTGCGCGAGGAATCAAACTGGCT‑GGGTTCAACGAGATTGGCTTGAAGACCTTGGGGTCTGACGATGCTGACATCAGTAGAGTGGCTATCGACCTCGTTCGCTCTCCTACTGGTATGCAGTCTGGTGCCTCAGGTAAGTTCGGTGCAACAGCTTATGACATCCATGAGAGACTTCATGGTACTGACCAGCGTACTTATAATGACTTGTACAAAGCAATATCTGACGCTATGAAAGACCCTGAGTTCTCTAc                     <  1:154974/301‑1 (MQ=255)`  
                                                                                                                                                                                                                                                                                      `aaTCAACCCTAAGACTCTAAACGAGTTCTCCGAGGTTGACCCTGAGAAGGCTGCGCGAGGAATCAAACTGGCT‑GGGTTCACCGAGATTGGCTTGAAGACCTTGGGGTCTGACGATGCTGACATCCGTAGAGTGGCTATCGACCTCGTTCGCTCTCCTACTGGTATGCAGTCTGGTGCCTCAGGTAAGTTCGGTGCAACAGCTTCTGACATCCATGAGAGACTTCATGGTACTGACCAGCGTACTTATAATGACTTGTACAAAGCAATGTCTGACGCTATGAAAGACCCTGAGTTCTCTACt                    >  1:149025/1‑301 (MQ=255)`  
                                                                                                                                                                                                                                                                                            `cccTAAGACTCTAAACGAGTTCTCCGAGGTTGACCCTGAGAAGGCTGCGCGAGGAATCAAACTGGCT‑GGGTTCACCGAGATTGGCTTGAAGACCTTGGGGTCTGACGATGCTGACATCCGTAGAGTGGCTATCGACCTCGTTCGCTCTCCTACTGGTATGCAGTCTGGTGCCTCAGGTAAGTTCGGTGCAACAGCTTCTGACATCCATGAGCGACTTCATGGTCCTGACCAGCGTACTTATAATGACTTGTACAAAGCAATGTCTGACGCTATGAAAGATCCTGAGTTCTCTAcctgtctc              >  1:82890/1‑294 (MQ=255)`  
                                                                                                                                                                                                                                                                                            `cccTAAGACTCTAAACGAGTTCTCCGAGGTTGACCCTGAGAAGGCTGCGCGAGGAATCAAACTGGCT‑GGGTTCACCGAGATTGGCTTGAAGACCTTGGGGTCTGACGATGCTGACATCCGTAGAGTGGCTATCGACCTCGTTCGCTCTCCTACTGGTATGCAGTCTGGTGCCTCAGGTAAGTTCGGTGCAACAGCTTCTGACATCCATGAGAGACTTCATGGTACTGACCAGCGTACTTATAATGACTTGTACAAAGCAATGTCTGACGCTATGAAAGACCCTGAGTTCTCTACTggcggc              >  1:206079/1‑301 (MQ=255)`  
                                                                                                                                                                                                                                                                                                   `cagctAAACGAGTTCTCCGAGGTTGACCCTGAGAAGGCTGCGCGAGGAATCAAACTGGCT‑GGGTTCACCGAGATTGGCTTGAAGACCTTGGGGTCTGACGATGCTGACATCCGTAGAGTGGCTATCGACCTCGTTCGCTCTCCTACTGGTATGCAGTCTGGTGCCTCAGGTAAGTTCGGTGCAACAGCTTCTGACATCCATGAGAGACTTCATGGTACTGACCAGCGTACTTATAATGACTTGTACAAAGCAATGTCTGACGCTATGAAAGACCCTGAGTTCTCTACTGGCGGCGCTAAGa       <  1:216273/298‑1 (MQ=255)`  
                                                                                                                                                                                                                                                                                                   `accctAAACGATTTCTCCGGTGTTGACCCTGAGAAGGCGGCGCGATGAATCAAACTGGCT‑TGGTTCACCGAGATTGGCTTGAAGACCTTGGGGTCTGACGATGCTGACATCCGTAGAGTGGCTATCGACCTCGTTCGCTCTCCTACTGGTATGCAGTCTGGTGCCTCAGGTAAGTTCGGTGCAACAGCTTCTGACATCCATGAGAGACGTCATGGTACTGACCAGCGTACTTATAATGACTTGTACAAAGCAATGTCTGACGCTATGAAAGACCCTGAGTTCTCTACTGGCGGCGCTAAGa       <  1:342381/298‑1 (MQ=255)`  
                                                                                                                                                                                                                                                                                                   `aCTCTGAACGAGTTCTCCGAGGTTGACCCTGAGAAGGCTGCGCGAGGAATCAAACTGGCT‑GGGTTCACCGAGATTGGCTTGAAGACCTTGGGGTCTGACGATGCTGACATCCGTAGAGTGGCTATCAACCTCGTTCGCTCTCCTACTGGTATGCAGTCTGGTGCCTCAGGTAAGTTCGGTGCAACAGCTTCTGACATCCATGAGAGACTTCATGGTACTGACCAGCGTACTTATAATGACTTGTACAAAGCAATGTCTGACGCTATGAAAGACCCTGAGTTCTCTACTGGCGGCGCTAAGa       <  1:275568/301‑1 (MQ=255)`  
                                                                                                                                                                                                                                                                                                   `aCTCTAAACGAGTTCTCCGAGGTTGACCCTGAGATGGCTGCGCGAGGAATCAAACTGGCG‑GGGTTCACCGAGATTGGCTTGAAGACCTTGTGGTCTGACGATGCTGACATCCGTAGAGTGGCTATCGACCTCGTTCGCTCTCCTACTGGTATGCAGTCTGGTGCCTCAGGTAAGTTCGGTGCAACAGCTTCTGACATCCATGAGAGACTTCATGGTACTGACCAGCGTACTTATAATGACTTGTACAAAGCAATGTCTGACGCTATGAAAGACCCTGAGTTCTCTACTGGCGGCGCTAAGa       <  1:201402/301‑1 (MQ=255)`  
                                                                                                                                                                                                                                                                                                    `ctctAAAAGAGTTCTCCGAGGTTGACCCTGAGAAGGCTGCGCGAGGAATCAAACTGGCT‑GGGTTCACCGAGATTGGCTTGAAGACCTTGGGGTCTGACGATGCTGACATCCGTAGAGTGGCTATCGACCTCGTTCGCTCTCCTACTGGTATGCAGTCTGGTGCCTCAGGTAAGTTCGGTGCAACAGCTTCTGACATCCATGAGAGACTTCATGGTACTGACCAGCGTACTTATAATGACTTGTACAAAGCAATGTCTGACGCTATGAAAGACCCTGAGTTCTCTACTGGCGGCGCTAAGAt      >  1:6110/1‑301 (MQ=255)`  
                                                                                                                                                                                                                                                                                                      `ctAAACGAGGTCTCCGAGGGTGACCATGAGAAGGCTGCGCGAGGAATCAAACTGGCT‑GGGTTCACCGAGATTGGCTTGAAGACCTTGGGGTCTGACGAGGCTGACATCCGTAGAGTGGCTATCGACCTCGTTCGCTCTCCTACTGGTATGCAGTCTGGTGCCTCAGGTAAGTTCGGTGCAACAGCTTCTGACATCCATGAGAGACTTCATGGTACTGACCAGCGTACTTATAATGACTTGTACAAAGCAATGTCTGACGCTATGAAAGACCCTGAGTTCTCTACTGGCGGCGCTAAGATGt    <  1:44691/301‑1 (MQ=255)`  
                                                                                                                                                                                                                                                                                                        `aaacGAGTTCTCCGAGGTTGACCCTGAGAAGGCTGCGCGAGGAATCAAACTGGCT‑GGGTTCACCGAGATTGGCTTGAAGACCTTGGGGTCTGACGATGCTGACATCCGTAGAGTGGCTATCGACCTCGTTCGCTCTCCTACTGGTATGCAGTCTGGTGCCTCAGGTAAGTTCGGTGCAACGGCTTCTGACATCCATGAGAGACTTCATGGTACTGACCAGCGTACTTATAATGACTTGTACAAAGCAATGTCTGACGCTATGAAAGACCCTGAGTTCTCTACTGGCGGCGCTAAGATGTcc  <  1:71594/301‑1 (MQ=255)`  
                                                                                                                                                                                                                                                                                                        `aaacGAGTTCTCCGAGGTTGACCCTGAGAAGGCTGCGCGAGGAATCAAACTGGCT‑GGGTTCACCGAGATTGGCTTGAAGACCTTGGGGTCTGACGATGCTGACATCCGTAGAGTGGCTATCGACCTCGTTCGCTCTCCTACTGGTATGCAGTCTGGTGCCTCAGGTAAGTTCGGTGCAACAGCTTCTGACATCCATGAGAGACTTCATGGTACTGACCAGCGTACTTATAATGACTTGTACAAAGCAATGTCTGACGCTATGAAAGACCCTGAGTTCTCTACTGGCGGCGCTAAGacgggc  >  1:158339/1‑296 (MQ=255)`  

                                                                                                                                                                                                                                                                                                           `|`                                                                                                                                                                                                                                                                                                            
`GTGTTTGGCGCAGGCATGTCTGCAATCAGTGACGCTGTAGCTGCTGGACTGAAACGCAGTAAACCAGAAGCTGAGTTCGACAATGAGTTCATCGGTCCTATGATGCGATTGGAAGCCCGTGAGACAGCACGAAACGCCAACTCTGCGGACCTCTCTCGGATGAACACTGAGAACATGAAGTTTGAAGGTGAACATAATGGTGTCCCTTATGAGGACTTACCAACAGAGAGAGGTGCCGTGGTGTTACATGATGGCTCCGTTCTAAGTGCAAGCAACCCAATCAACCCTAAGACTCTAAAAGAGTTCTCCGAGGTTGACCCTGAGAAGGCTGCGCGAGGAATCAAACTGGCT‑GGGTTCACCGAGATTGGCTTGAAGACCTTGGGGTCTGACGATGCTGACATCCGTAGAGTGGCTATCGACCTCGTTCGCTCTCCTACTGGTATGCAGTCTGGTGCCTCAGGTAAGTTCGGTGCAACAGCTTCTGACATCCATGAGAGACTTCATGGTACTGACCAGCGTACTTATAATGACTTGTACAAAGCAATGTCTGACGCTATGAAAGACCCTGAGTTCTCTACTGGCGGCGCTAAGATGTCC  >  NC_001604.1/31780‑32376`

  

Alignment Legend

Aligned base mismatch/match (shaded by quality score): `ATCG/ATCG` `< 3 ≤` `ATCG/ATCG` `< 11 ≤` `ATCG/ATCG` `< 25 ≤` `ATCG/ATCG` `< 36 ≤` `ATCG/ATCG` `< 38 ≤` `ATCG/ATCG`

Unaligned base: `atcg`    Masked matching base: `atcg`    Alignment gap: `‑`    Deleted base: `‑`
